# Continual Learning Strategy in One-Stage Object Detection Framework Based on Experience Replay for Autonomous Driving Vehicle

**DOI:** 10.3390/s20236777

**Published:** 2020-11-27

**Authors:** Jeng-Lun Shieh, Qazi Mazhar ul Haq, Muhamad Amirul Haq, Said Karam, Peter Chondro, De-Qin Gao, Shanq-Jang Ruan

**Affiliations:** 1 Department of Electronic and Computer Engineering, National Taiwan University of Science and Technology, Taipei 106, Taiwan; m10802127@mail.ntust.edu.tw (J.-L.S.); d10702803@mail.ntust.edu.tw (Q.M.u.H.); d10902802@mail.ntust.edu.tw (M.A.H.); m10802818@mail.ntust.edu.tw (S.K.); 2 Information and Communications Research Laboratories, Embedded Vision and Graphics Technology Department, Division for Embedded System and SoC Technology, Industrial Technology Research Institute, Hsinchu 31057, Taiwan; peterchondro@itri.org.tw (P.C.); charliegao@itri.org.tw (D.-Q.G.)

**Keywords:** continual learning, one-stage object detection, autonomous driving vehicles

## Abstract

Object detection is an important aspect for autonomous driving vehicles (ADV), which may comprise of a machine learning model that detects a range of classes. As the deployment of ADV widens globally, the variety of objects to be detected may increase beyond the designated range of classes. Continual learning for object detection essentially ensure a robust adaptation of a model to detect additional classes on the fly. This study proposes a novel continual learning method for object detection that learns new object class(es) along with cumulative memory of classes from prior learning rounds to avoid any catastrophic forgetting. The results of PASCAL VOC 2007 have suggested that the proposed ER method obtains 4.3% of mAP drop compared against the all-classes learning, which is the lowest amongst other prior arts.

## 1. Introduction

A deep learning algorithm is expected to learn from data that are always changing. Consequently, a deep learning model is required to be trained repeatedly from the ground-up to stay relevant. In nature, humans are capable of learning new things continuously while retaining their previous knowledge. This ability to learn new things continuously is also known as continual or life-long learning. In contrast, for a machine, especially a deep learning model, learning new things naïvely means overwriting its previous knowledge. Instead of learning additional knowledge, the model will lose its ability to detect previous class(es). However, naïvely forcing a model that is already trained to learn additional classes without any strategy can lead to catastrophic forgetting. Catastrophic forgetting is a phenomenon in which a model forgets the previously learned knowledge. This forgetting of the previous knowledge is a major shortcoming of the convolutional neural networks (CNN). In order to preserve the performance on previous knowledge, a training strategy is needed to overcome the shortcoming of the naive training while learning additional classes [1]. Continual learning challenge has persisted for decades in the deep learning field. Moreover, as new data with more classes have become easier to obtain in recent years, continual learning has gained more attention from the research community [2,3].

**Figure 1:**
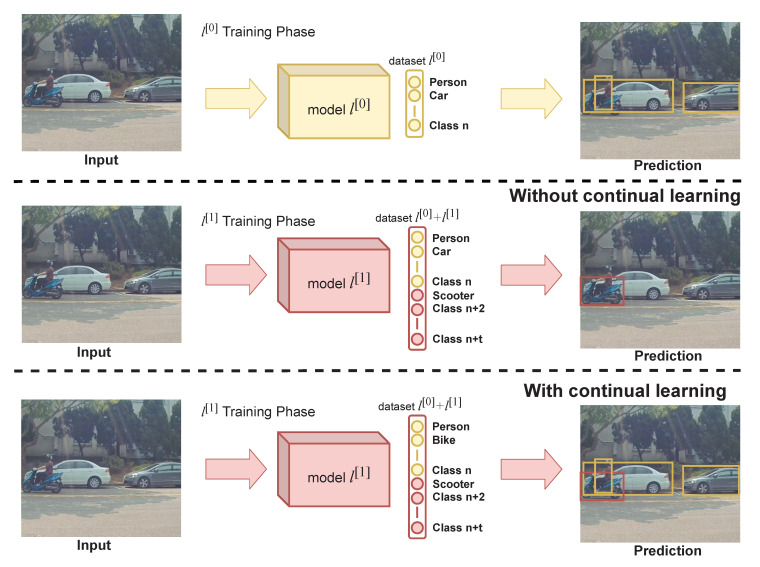
Continual learning purpose is to increase the number of classes a model can detect while prevents overwriting of previous knowledge.

The phenomenon of catastrophic forgetting is illustrated in Figure 1. Suppose that a model $l^{\left[0\right]}$ is capable of detecting *n* number of classes in $l^{\left[0\right]}$ dataset, then given a certain condition, the model is required to detect *t* additional classes in $l^{\left[1\right]}$ dataset, as illustrated in Figure 1. If in the model $l^{\left[1\right]}$ training, for the purpose of detecting these *t* number of additional classes, the dataset that is provided only contains the (*n* + 1)th to *t*th classes without providing previously learned (1st to *n*th) classes’ dataset, model $l^{\left[1\right]}$ will forget these previously learned classes. As a result the model $l^{\left[1\right]}$ loses its ability to detect any object from dataset $l^{\left[0\right]}$. This phenomenon happens because weights and biases of previously learned classes get completely overwritten when training for additional classes. Any research attempt at dealing with catastrophic forgetting is also known as continual learning. Generally, continual learning is represented as incremental learning and active learning in the research literature.

In this era of increasing data, the autonomous driving vehicle (ADV) datasets are also developing. Thus, the complexity of deep learning application for ADV has increased with the introduction of novel vehicles and traffic signs. Therefore, it is expected that existing models could be updated to detect new classes [4,5,6,7,8]. Furthermore, the public dataset available on the Internet may not share the same classes and characteristics due to the distinctive driving environment and vehicles in a different country. For instance, in mainstream ADV datasets that are publicly accessible like KITTI [9] and Cityscape [10], some unique classes available in Taiwan, such as Scooter, can not be found in those datasets. For generic classes presented in public datasets, the developer may be able to obtain a pre-trained model with high accuracy trained on these public datasets. Nevertheless, this high-performance model still needs to be trained again to detect certain classes that are not present in other public datasets. Thus, it becomes increasingly urgent to increase the number of classes that our model can detect without risking the drop in accuracy on the previously learned classes by utilizing continual learning in ADV.

Numerous researches have been proposed to deal with catastrophic forgetting. However, most of those solutions only focus on the classification problem while the problem of catastrophic forgetting on object detection is still largely untouched [11,12,13,14]. Even though one can attach an object proposal algorithm before the classification network to create an object detection framework, the resulting object detection framework would be considered as a two-stage detection framework. However, this framework is not suitable for real-time application because of its high inference time [1]. In gradient episodic memory (GEM) [15], previous data are stored in an episodic memory to avoid forgetting previous knowledge in the current continual training. They offer forward and backward transfer of knowledge. However, the backward transfer is essential for the previous task that increases the computation time. Moreover, they used a gradient constraint approach. This approach limits the gradient to prevent the weight from being drastically changed. While such a strategy is able to preserve the weights to some degree, it also limits the network’s ability to learn new classes effectively. Consequently, this strategy often faces a dilemma where it should choose between preserving the previously learned classes’ weights or radically changes its weights to learn new classes effectively. In recent years, a new strategy which employs knowledge distillation (KD) [16] that is originally utilized for model compression, has been proposed as an alternative [1,17]. While this strategy is generally more stable and yields better results than gradient constraint, it demands huge additional data that should be similar to previous classes. This additional data requirement highly affects the performance of the model. Thus, it defeats the original purpose of continual learning to add scarce and unique classes incrementally.

To overcome the shortcoming of prior works, a method that mimics the biological system’s ability to replay past experiences is proposed. A similar strategy has been implemented successfully in image classification [15] and reinforcement learning [18]. Nevertheless, object detection is a completely different domain. Several modifications and adjustments have to be made for the proposed method to work properly, mainly on the issues concerning memory integration during $l^{\left[1\right]}$ training phase. Our research has demonstrated that replay can be used as an effective strategy in continual learning. In this paper, the proposed method is demonstrated in You-Only-Look-Once (YOLO) since it is one of the most popular one-stage detection framework [19]. However, the proposed method is flexible and can be implemented in other object detection frameworks without modifying the network architecture.

## 2. Related Work

Even though there has been plenty of work addressing continual learning, they typically only focused on the classification problem. This research focuses on the more practical setting in the context of object detection. There has been less research on continual learning in object detection scenarios. Amidst those few is the continual learning scheme applied to faster region convolution neural networks (Faster R-CNN) [1,20] object detection framework. They avoid catastrophic forgetting of previously learned knowledge by distilling knowledge from the previous model. The external distilled proposals of the prior network are saved and utilized as pseudo-data in continual learning. They freeze previous network layers while training the continual one. The whole network layers are utilized at the inference stage. However, Faster R-CNN is a two-stage object detector that consists of an external network for extracting proposals that result in higher computational complexity. Another recent approach to overcome the catastrophic forgetting is proposed in [21], that improved the learning procedure of EWC for object detection. To remember the previous knowledge, they proposed a pseudo-annotation of previously learned classes. A Laplace approximation [22] is proposed for the likelihood of each task to be diagonal.

Other research that focuses on continual learning on object detection is deep model consolidation (DMC) [17]. In the DMC, a double distillation loss has been proposed to combine two models that specialize in different classes into one compact model that can detect all the preceding models’ classes. First, they trained two networks on different data. Then, they consolidated both models into one single model using double distillation loss through training on unlabeled auxiliary data. Although DMC is fast at the inference stage, the training time is extremely long compared to baseline object detectors and demands higher computational power since the consolidation phase requires three models to run simultaneously. Moreover, additional data needed for the consolidation phase are massive since they are not labeled. Furthermore, auxiliary data needed for the consolidation phase highly affect the performance of the consolidated model. Thus, results will vary according to the number of images and domain similarity between the original and auxiliary data. Therefore, in a case where many unique and rare classes are involved, DMC may not perform very well.

Other prior works regarding continual learning methods for object detection are inspired by KD [16], where the previous knowledge is saved in the frozen copy of the previous model. Moreover, the object detection frameworks considered for knowledge distillation are proposal generation-based methods and utilizing stored proposals from previous tasks [1]. Some models are based on auxiliary data and multi-models training as proposed in DMC [17] that consumes more computational power and resources. In contrast, the proposed approach is more general and can be used in any conventional object detection models. The proposed approach does not require any auxiliary data while learning the current data. A comprehensive comparison between the advantages and disadvantages of the proposed method and previous methods are described in Section 4.

## 3. Proposed Methodology

**Figure 2:**
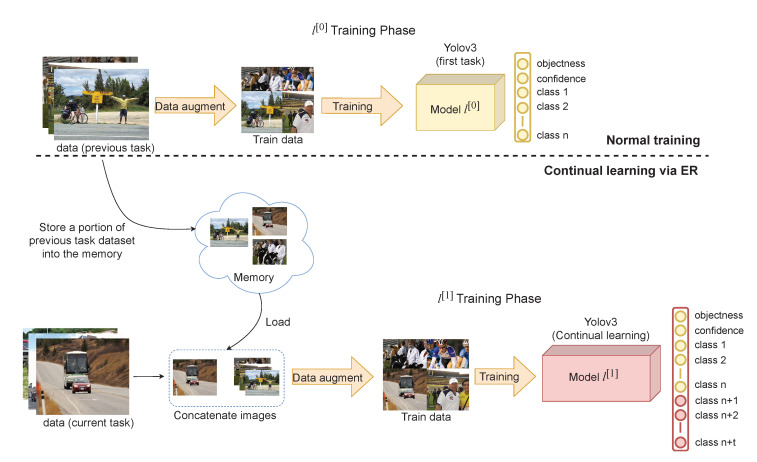
Block diagram of the proposed continual learning strategy method.

Suppose that a model $l^{\left[0\right]}$ is trained normally on *n* number of classes from the first task dataset that will be referred to as $l^{\left[0\right]}$ dataset, then the model $l^{\left[0\right]}$ is needed to detect additional *t* number of classes without using the whole data from the $l^{\left[0\right]}$ dataset. To avoid catastrophic forgetting where the model forgets features learned in classes belonging to $l^{\left[0\right]}$ dataset, a method which utilizes memory is proposed. The proposed method, which will be referred to as Experience Replay (ER), works by saving a portion of the $l^{\left[0\right]}$ dataset into the memory. Then, the images in the memory will be concatenated with the second task dataset, denoted as $l^{\left[1\right]}$ dataset in every iteration during $l^{\left[1\right]}$ training phase as shown in Figure 2. Furthermore, the dynamic omission is implemented in order to ensure that the memory and $l^{\left[1\right]}$ dataset can be adequately combined during $l^{\left[1\right]}$ training phase.

### 3.1. YOLO Architecture

**Figure 3:**
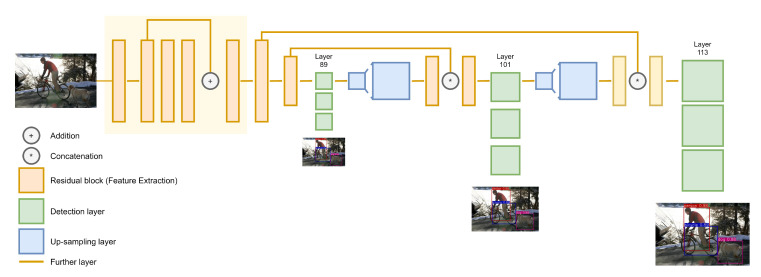
Architecture of YOLOv3 with DarkNet53 as target network for the proposed continual learning strategy.

The proposed ER method utilizing YOLO is suitable for real-time applications. Nevertheless, the proposed ER method is a flexible strategy and easy to implement in other frameworks without modifying the architecture. The architecture of YOLO that is utilized can be seen in Figure 3. To predict objects’ location, YOLO utilizes grid cells with a size of N×N with three different scales located at layer 89, 101, and 113. Each grid cell has a fixed number of predictions depending on the number of anchor boxes specified. To update YOLO’s weight, the loss between the target and model prediction needs to be calculated. First, in each grid cell, the bounding box regression loss ($L_{bb}$) is calculated using generalized intersection over union (GIOU) [23]. Suppose that a target bounding box, which is denoted as Tx, and a predicted bounding box denoted as Ty, both of which contain the coordinate and size of bounding boxes. Meanwhile, the algorithm can calculate the value of *C* by measuring the minimum rectangular area that surrounds both bounding boxes. Thus, the algorithm can get the GIOU by using Equation (1). Then, the resulting value is used as the input for Equations (3) and (5).

Aside from coordinates and sizes, each bounding box also contains an objectness score, which is denoted as Xo and Yo for predicted and target bounding boxes, respectively. The objectness score of a predicted bounding box can measure between 0 and 1, whereas for target bounding boxes, the score is only 0 or 1 depending on the target object’s presence in the corresponding grid cell. Meanwhile, the predicted and target bounding boxes’ classification scores are denoted as Xc and Yc, respectively. However, unlike the objectness score with a single value for each bounding box, the confidence score is represented as a one-hot vector with a length equal to the number of the model’s classes. The confident score of each bounding box is the multiplication of objectness and classification scores, as shown in Equations (4) and (5), respectively. Both objectness and classification losses are calculated using binary cross-entropy (BCE) as written in Equation (2), whereas Cx and Cy represent the input and output of the function, respectively. It is important to note that the input of BCE should be normalized using the sigmoid function (σ) to avoid an exploding gradient. In Equation (2), *w* denotes the positive weight which should be used when there is a drastic imbalance between precision and recall. If there is no problem with the imbalance, the algorithm uses the default value of *w*, which is 1. Lastly, the total loss function, as shown in Equation (7) is calculated by adding $\alpha \times L_{conf}$ and $\beta \times L_{bb}$, with α and β denote the weight for each of their respective loss.

(1) $$GIOU\left(Tx,Ty\right)=\frac{\left|Tx\cap Ty\right|}{\left|Tx\cup Ty\right|}-\frac{\left|C\backslash (Tx\cup Ty)\right|}{\left|C\right|},$$

(2) $$BCE\left(Cx,Cy\right)=-w\left[Cy\times \log\sigma (Cx)+(1-Cy)\times \log(1-\sigma (Cx))\right],$$

(3) $$L_{bb}\left(Xt,Yt\right)=\frac{1}{N^{2}}\sum_{i,j=1}^{N}1-GIOU\left(Xt_{ij},Yt_{ij}\right),$$

(4) $$L_{cls}\left(Xc,Yc\right)=\frac{1}{N^{2}}\sum_{i,j=1}^{N}BCE\left(Xc_{ij},Yc_{ij}\right),$$

(5) $$L_{obj}\left(Xo,Yo\right)=\frac{1}{N^{2}}\sum_{i,j=1}^{N}BCE\left(Xo_{ij},GIOU\left(Xo_{ij},Yo_{ij}\right)\right),$$

(6) $$L_{conf}=L_{obj}\times L_{cls},$$

(7) $$Loss=\left(\alpha \times L_{conf}\right)+\left(\beta \times L_{bb}\right).$$

### 3.2. Task Distribution and Data Augmentation

The Pascal VOC 2007 [24] dataset is used for training the proposed ER method. For experiment purposes, the dataset is divided into the $l^{\left[0\right]}$ and $l^{\left[1\right]}$ tasks. The $l^{\left[0\right]}$ dataset is used to train YOLO normally, whereas the $l^{\left[1\right]}$ dataset is used for continual learning. The purpose of training on the $l^{\left[1\right]}$ dataset is to simulate a real-world condition where the model is needed to learn additional classes. The $l^{\left[1\right]}$ dataset has entirely different classes from the $l^{\left[0\right]}$ dataset. After the model $l^{\left[0\right]}$ is trained normally using the $l^{\left[0\right]}$ dataset, it will be used as the pre-trained weight for the continual learning on the $l^{\left[1\right]}$. It is also important to increase the variance of training data. By utilizing data augmentation correctly, the accuracy of the model can be further improved as elaborated in Section 4.2.

The most recent YOLO utilizes Cut-Mix [25] for data augmentation. In ER implementation for the task $l^{\left[1\right]}$, Cut-Mix should be applied after images are loaded and concatenated with the new dataset. Saving augmented images into memory instead of non-augmented ones can slightly decrease the accuracy as shown in our experiment in Section 4. Another important thing to note is the effect of the imbalance number of data between the $l^{\left[0\right]}$ and $l^{\left[1\right]}$ dataset, which is shown in Table 1. In the 19 + 1 scheme, the purpose of switching between TV and person classes as the $l^{\left[1\right]}$ task is to observe this particular issue. The imbalance data between $l^{\left[0\right]}$ and $l^{\left[1\right]}$ tasks may provide a better understanding of how to implement ER correctly.

### 3.3. Memory Replay on Continual Learning

**Figure 4:**
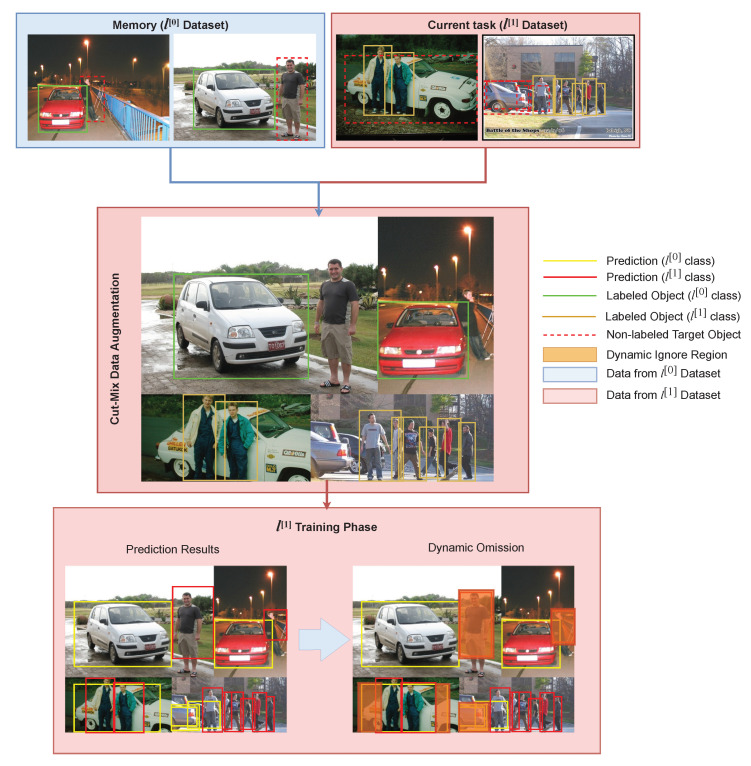
Dynamic omission prevents models from training recklessly from predictions that have no corresponding labels.

Given a training with *b*-size batch, each iteration will contain *c*-number of training images from the $l^{\left[1\right]}$ dataset and (b−c) images from the $l^{\left[0\right]}$ dataset. Then, images from the $l^{\left[0\right]}$ dataset and $l^{\left[1\right]}$ dataset are concatenated. By mixing images from the $l^{\left[0\right]}$ dataset, the model would be able to avoid catastrophic forgetting. However, this strategy causes the model to train longer since each iteration only contains *c*-number of images from the $l^{\left[1\right]}$ task dataset. Therefore, one training epoch will have the same number of iterations as the number of images in the new dataset divided by *c*, regardless of the batch size. Details of the proposed continual learning algorithm is described in Algorithm 1, specifically in line 12–23. When training the model $l^{\left[1\right]}$ in the continual learning scheme, the loss function is the same as the one used in normal training. The $L_{conf}$ is the multiplication result of $L_{cls}$ and $L_{obj}$ which are calculated using BCE in Equation (6), whereas for $L_{bb}$ the loss function used is the GIOU loss as shown in Equation (3). During the $l^{\left[1\right]}$ training phase, the $L_{obj}$ is affected by dynamic omission, which is described as in Algorithm 1 from line 21 to 27. Suppose a bounding box is predicted as a class that belongs to $l^{\left[0\right]}$ dataset, then the $L_{obj}$ of the corresponding bounding box is invalidated as it may happen because of an unlabeled target object. This rule is to prevent reckless training, which is illustrated in Figure 4. Reckless training occurs since the pre-trained model that is used in $l^{\left[1\right]}$ training phase is already capable of predicting objects which belong to $l^{\left[0\right]}$ dataset. Thus, when training $l^{\left[1\right]}$ model, it may detect an object which belongs to $l^{\left[0\right]}$ dataset in the $l^{\left[1\right]}$. Conversely, the opposite occurrence, where the model detects objects that belong $l^{\left[1\right]}$ dataset in the memory, can also occur. However, since labels of $l^{\left[0\right]}$ objects are not present in $l^{\left[1\right]}$ dataset and contrariwise, calculating losses from these predictions is inadvisable. It is important to note that this strategy is also highly affected by the number of images from $l^{\left[0\right]}$ dataset denoted as *m* that is stored in the memory. It is not compulsory to store all images from $l^{\left[0\right]}$ dataset into the memory. However, having more images in the memory can lead to better accuracy for $l^{\left[0\right]}$ classes.
**Algorithm 1** The proposed continual learning strategy
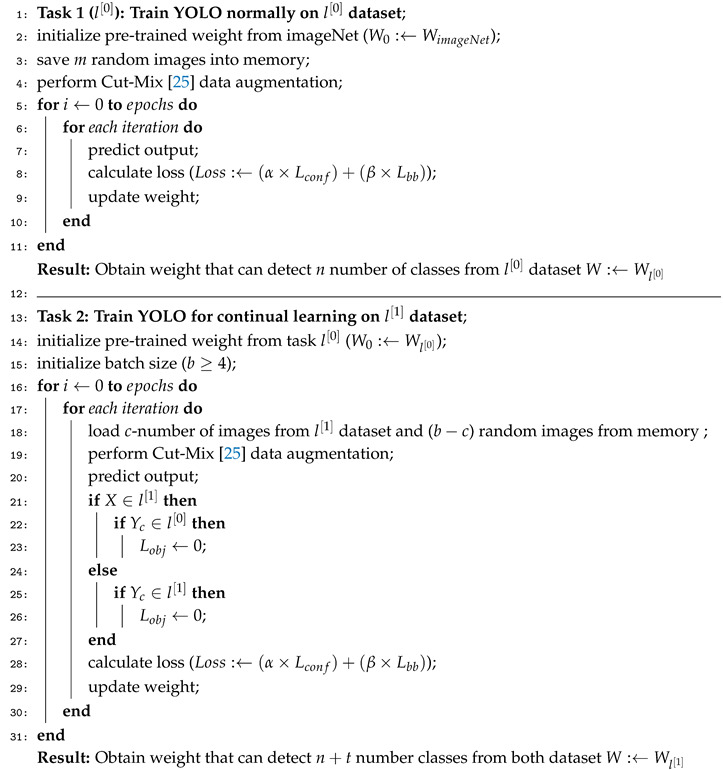


## 4. Results

In this section, the proposed ER method is extensively evaluated on Pascal VOC 2007 dataset. Results are provided for three different modifications to the proposed ER method. ER for 2500 memory size, ER for 2500 memory size with data augmentation, and ER for 1000 memory size with data augmentation. The proposed ER method is implemented on NVIDIA 1080 GPU. The proposed ER method is trained using stochastic gradient descent (SGD) [26] for 100 iterations. All experiments are written in Python using Pytorch [27] as the machine learning framework. Parameters for training the model is described in Table 2.

**Figure 5:**
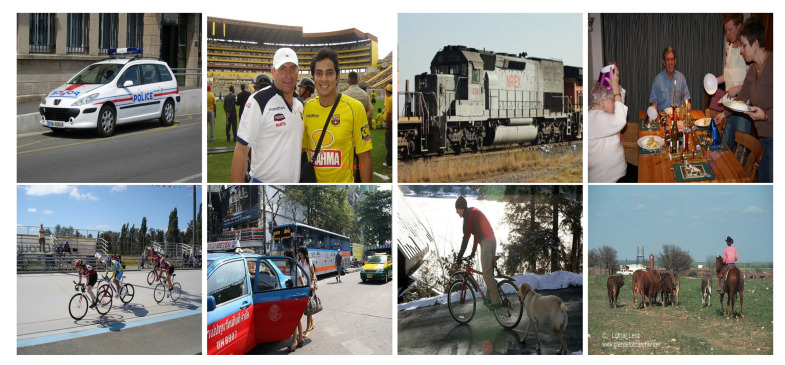
Sample images from the pascal VOC 2007 dataset.

**Figure 6:**
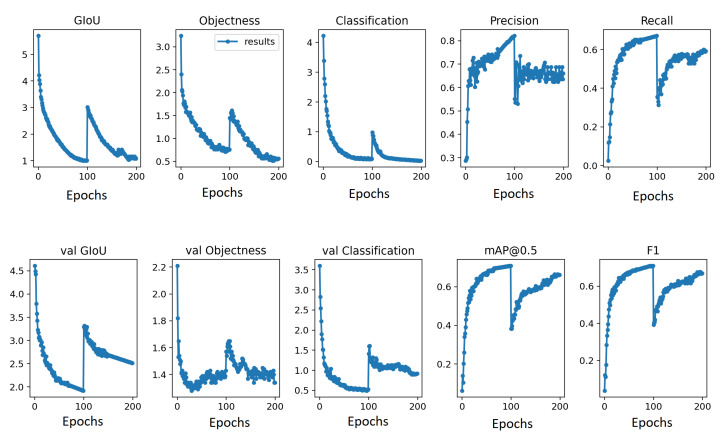
Training details for previous and current task. The *x*-axis represents epochs in which epoch 0–99 is the training on $l^{\left[0\right]}$ task, whereas epoch 100–199 is the training on $l^{\left[1\right]}$ task.

The distribution of the *trainval* and the *test* set for training and testing, respectively, is unchanged from the Pascal VOC 2007 official release. Following experiments presented in [1,17], the proposed continual learning method is evaluated on two tasks such that ten classes for the $l^{\left[0\right]}$ task and ten classes for $l^{\left[1\right]}$ or the continual task. The other experiment is performed on 19 classes for $l^{\left[0\right]}$ task and 1 class for $l^{\left[1\right]}$ task. The distribution of objects and images for 10 + 10 classes and 19 + 1 classes are presented in Table 1 in Section 3. A few images of the Pascal VOC 2007 are visualized in Figure 5. The graphs of loss, classification, recall, precision, and mean average precision (mAP) for both training such that the previous task and the current task are shown in Figure 6.

### 4.1. Performance Evaluation Parameters

The mAP is calculated independently for the $l^{\left[0\right]}$ and $l^{\left[1\right]}$ tasks. To calculate the mAP, the precision and recall score have to be obtained first from the discrepancy between ground-truth and prediction. Precision, also known as sensitivity, is the ratio of true positives to the sum of true positives and true negatives. Recall, also known as specificity, is the ratio of true positives to the total number of true positives and false negatives. Formulas for the precision and recall is given in Equations (8) and (9) respectively. The AP and mAP are calculated in Equations (10) and (11). A Jaccard index (J) is used to calculate the overlap area between predicted and ground-truth bounding box *A* and *B*, as shown in Equation (12).

(8) $$Precision=\frac{TP}{TP+TN},$$

(9) $$Recall=\frac{TP}{TP+FN},$$

(10) $$AP=\int_{0}^{1}p\left(r\right)dr,$$

(11) $$mAP=1/N\sum_{i=1}^{N}AP_{i},$$

(12) $$J\left(A,B\right)=\frac{\left|A\cap B\right|}{\left|A\cup B\right|},$$

where AP, TP, and FP denotes average precision, true positive, and false positive, respectively, whereas p(r) represents the probability of the event under the precision and recall curve. The mAP is the mean of AP for *N* number of classes. The proposed ER method is compared with prior state-of-the-art continual learning methods [1,15,17] for object detection. These methods have been implemented on different object detection framework by respective authors. However, for a fair comparison, these methods are adopted to the YOLO framework. Results of these adopted methods are then compared with the proposed ER method.

### 4.2. Addition of Classes Incrementally

In the first experiment, consider the first 19 classes that appear in alphabetical order in Pascal VOC 2007 dataset as the $l^{\left[0\right]}$ task, and the remaining one as the $l^{\left[1\right]}$ task. The model $l^{\left[0\right]}$ is trained normally on the first (1–19) classes on *trainval* subset, and the model $l^{\left[1\right]}$ is trained using continual strategies on the remaining one class, which is the TV class. A summary of the comparison between the proposed ER method and state-of-the-art methods are shown in Table 3. The baseline approach for continual learning is to store some of the images from $l^{\left[0\right]}$ task in memory and replay that memory repeatedly while training on $l^{\left[1\right]}$ task. By replaying those memories, ER is able to preserve features of classes in the $l^{\left[0\right]}$ dataset. Furthermore, it maintains the same accuracy on $l^{\left[1\right]}$ classes. In the $l^{\left[0\right]}$ training phase, 19 classes are trained first. Then during the $l^{\left[1\right]}$ training phase, only one class is incrementally trained. This scheme is performed to observe the effect of class distribution during continual learning. In comparison to previous methods [1,15,17], the proposed ER method has significantly higher mAP for all classes. The proposed ER method have increased the mAP to 8.9%, 8.8%, and 30.5% in comparison to GEM [15], DMC [17], and KD [1] respectively. Specifically, the proposed ER method with data augmentation has achieved 68.9% mAP for all 20 classes.

The second experiment is performed on the 10 + 10 class scheme. The first ten classes (1–10) are trained as the $l^{\left[0\right]}$ task, and the remaining ten (11–20) are trained as $l^{\left[1\right]}$ task for continual learning. As presented in Table 4, GEM is the only method that achieves a higher result than the proposed ER method, yet only for the chair class. The reason behind this is the imbalanced data in the Pascal VOC 2007 dataset. The proposed ER method with 1000 memory size has slightly higher results than the 5000 memory size for $l^{\left[0\right]}$ classes (11–20). However, the proposed ER method for 5000 memory size with augmentation has improved the mAP for all classes up to 65.5%. In the 10 + 10 class scenario the proposed ER method has increased the mAP up to 54.3%, 30%, and 14.6% as compared to GEM [15], KD [1], and DMC [17] respectively. To better investigate the proposed ER method’s continual learning behaviour, an experiment is performed on the 19 + 1 scheme with the person class as the incremental class instead of TV. Table 5 presents experiments performed on normal training (1–19), normal training (20), GEM, and the proposed ER method. As shown in Table 5, the mAP for the normal training is 68.8% while the proposed ER method achieved 67.1%. In comparison to GEM, the proposed ER method has 15.2% increased the mAP for all classes. Results presented in Table 5 indicates that the proposed ER method performed better in preserving features of $l^{\left[0\right]}$ task.

### 4.3. Visualization and Effect of Different Memory Size

**Figure 7:**
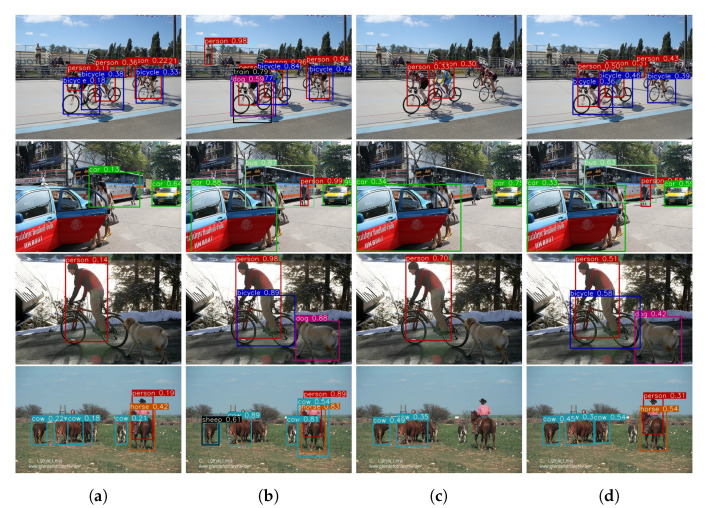
Object detection performance of the proposed ER method with KD [1], DMC [17], GEM [15], and the proposed ER method on test dataset as shown above from (**a**–**d**) respectively.

In order to provide an insight of the proposed ER method’s performance compared with prior methods, several prediction results are visualized from the Pascal VOC 2007 dataset. Those images are obtained from the *test* set that contain both the $l^{\left[0\right]}$ and $l^{\left[1\right]}$ classes. The performance of each method is visualized in Figure 7. Images from Figure 7a–d presents the object detection performance on KD [1], DMC [17], GEM [15], and the proposed ER method respectively. In Figure 7a KD [1] and Figure 7c GEM [15] are shown to have many false negatives. Generally, KD [1] has comparatively better performance on the $l^{\left[1\right]}$ task in comparison to $l^{\left[0\right]}$ task. Note that, the person class belongs to the $l^{\left[1\right]}$ task whereas the car class belong to the $l^{\left[0\right]}$ task. The same also happens with GEM [15], since it fails to recognise the bicycle class which belongs to $l^{\left[0\right]}$ task. Meanwhile, the performance of DMC [17] as shown in Figure 7b is considerably better than KD [1] and GEM [15]. However, DMC [17] has more false positive than other methods which are visualized as black bounding boxes such as train and dog in Figure 7a first row. This false positives occur because the mean of DMC’s confidence score is higher than other methods. In comparison to all these prior methods, the proposed ER method which utilizes 2500 memory as shown in Figure 7d has correctly localized and classified all objects. However, as shown in Figure 7d first row, far objects are not detected by the proposed ER method. The false negative of far objects is the limitation of this work.

**Figure 8:**
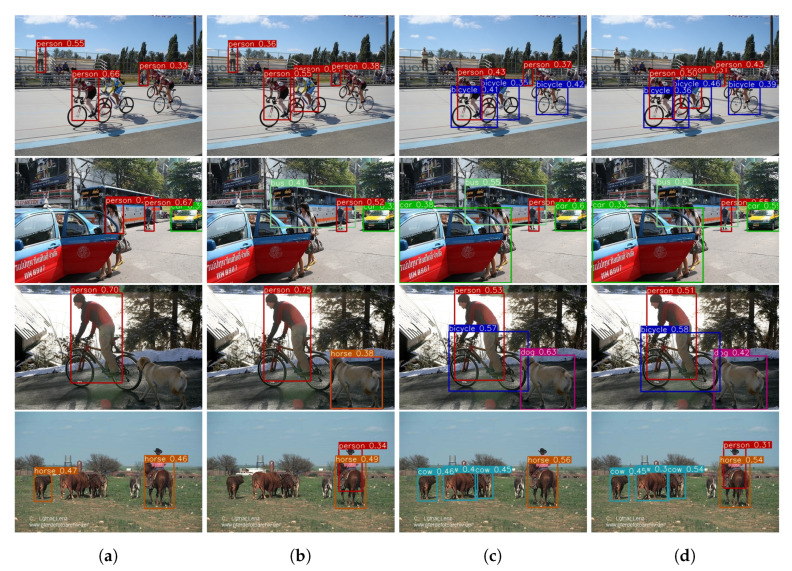
Prediction of the proposed ER method with different memory size, (**a**) memory size of 500 frames, (**b**) memory size of 1000 frames, (**c**) memory size of 2500 frames, and (**d**) memory size of 5000 frames.

In Figure 8, four identical images as the one presented in Figure 7 have been considered for prediction using ER in various memory size to provide better comprehension of the effect of different memory size. In order to evaluate the effect of memory size on results, different experiments are performed on 500, 1000, 2500, and 5000 memory size in the 10 + 10 class scenario. As shown in Table 6, the difference between the mAP for the memory size 5000 and 2500 are 0.1%. However, there is 4.3% accuracy drop for the 1000 memory size. The proposed ER method has comparable results in all the memory size. As the memory size is increased, the capability of the model to predict many objects also increases. However, the results of memory size 2500 and 5000 are relatively similar with only 0.1% difference in the average mAP. The fewer memory size requires fewer training time and hardware’s memory requirement. However, it will reduce the performance on $l^{\left[0\right]}$ classes.

### 4.4. Performance Evaluation on ITRI-DrvieNet60 Dataset

The proposed ER model is trained on the ITRI-DriveNet-60 private dataset. ITRI-DriveNet-60 private dataset is taken on the highways of Taiwan. It has similar characteristics with famous autonomous driving object detection dataset such as KITTI [9] and Cityscape [10]. The number of images and objects for *train* and *test* set are shown in Table 7. Four classes (four-wheel vehicle, rider, two-wheel vehicle, and person) are introduced in this dataset. The task distribution strategy for the ITRI-DriveNet-60 dataset’s continual learning is presented in Table 8. We trained the model on the 1 + 3 classes scheme. Therefore, the four-wheel vehicle class is considered in $l^{\left[0\right]}$ training, and the other classes are not considered in $l^{\left[0\right]}$ training those are represented by dashes in Table 8. and the other three classes are considered in $l^{\left[1\right]}$ training. While training the $l^{\left[1\right]}$ classes, the $l^{\left[0\right]}$ class labels are ignored and not utilized for training. The AP for the normal training, $l^{\left[0\right]}$ training, and $l^{\left[1\right]}$ training are illustrated in Table 8. Notably, the single class accuracy is higher because it is easier for the model to classify a single class. However, in the $l^{\left[1\right]}$ training, the four-wheel vehicle class accuracy is dropped to 4.5% still the four-wheel vehicle accuracy is higher among other classes. The total accuracy of four-wheel vehicle class in the $l^{\left[1\right]}$ training is 85.2%. The mAP of the proposed method is almost similar to the mAP of the normal training. Specifically, the proposed method obtains 77.1% mAP for all four classes. The mAP drops only 0.1% in comparison to normal training on the YOLOv3 object detection framework. This indicates the effectiveness of the proposed ER method for continual learning. Conclusively, the proposed ER method achieved comparably the same accuracy as the normal training.

**Figure 9:**
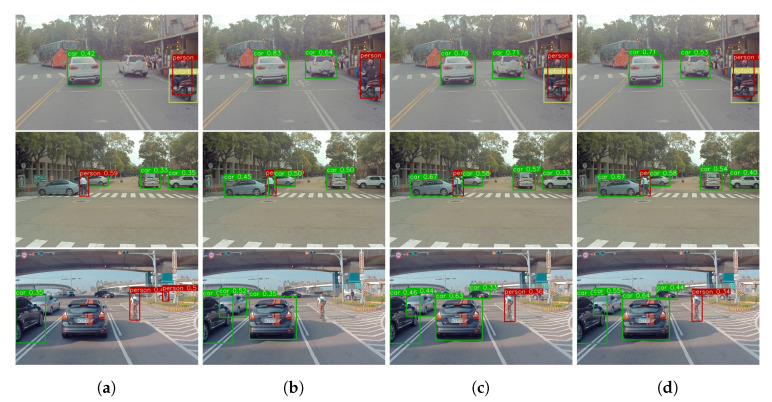
The object detection result on ITRI-DriveNet-60 private dataset on various memory size, (**a**) memory size of 500 frames, (**b**) memory size of 1000 frames, (**c**) memory size of 2500 frames, and (**d**) memory size of 5000 frames.

The proposed ER method has excellent detection results when using the higher memory size (2500 and 5000 frames) as shown in Figure 9c,d respectively. On the other hand, the fewer memory size (500 and 1000 frames) has a tendency to detect one of the tasks better than the other. As illustrated in Figure 9a the model with memory size 500 frames can detect classes which belong to $l^{\left[1\right]}$ dataset better than classes which belong to $l^{\left[0\right]}$ dataset, whereas the opposite is true for the model with memory size 1000 frames. This condition occurs because employing a memory size of 1000 frames will preserve more features from the $l^{\left[0\right]}$ dataset compared with only utilizing a memory size of 500 frames. However, using higher memory will results in better performance from both $l^{\left[0\right]}$ and $l^{\left[1\right]}$ dataset since more images in memory can be shuffled, resulting in more variance while training. It is notable from Figure 9d that the memory size 5000 frames have better results among others. However, similar to prediction results in Figure 8, the detection result of the memory size 2500 and 5000 frames are comparable. This result indicates that even utilizing ER with the fewer memory size can achieve acceptable performance regardless of the dataset, as proven in Table 6 and Table 8 which are performed in two different datasets.

Continual learning strategies tend to have higher training time compared with normal training. In normal training, after the model is run in inference for obtaining the prediction, backpropagation is performed to update the weights based on the loss between prediction and ground-truth. However, the prior works typically run the $l^{\left[0\right]}$ or previous model alongside the $l^{\left[1\right]}$ model and use the $l^{\left[0\right]}$ model prediction to learn previous features (KD [1] and DMC [17]) or compare the gradients of both models (GEM [15]). These approaches are rather cumbersome and take a longer time compared with normal training. Therefore, in the proposed ER method, a straight-forward approach has been considered, which is able to reduce the training time. In the proposed ER method, the noticeable addition in each iteration’s training time is caused by the integration and augmentation of the memory in the $l^{\left[1\right]}$ training phase, which has an average of 200 ms for each iteration as described in Table 9. Meanwhile, the dynamic omission added to the algorithm is very simple. Thus, its time consumption is negligible. Table 9 also presents the training time of each process in the prior works. Based on these processes, the training time for a single iteration is calculated as the sum of all these processes. The average training time for the proposed ER methods is shown to be the lowest among other continual strategies. Table 10 provides the advantages and disadvantages of the proposed ER method in comparison to the previous methods. The proposed ER method achieves better detection results in comparison to other continual learning methods while having the lowest time complexity.

## 5. Conclusions

In this research, a novel method to address the problem of catastrophic forgetting is proposed. Using YOLO architecture as the benchmark framework, ER can preserve the features from $l^{\left[0\right]}$ task while training on the $l^{\left[1\right]}$ task. A guide to implementing the data augmentation technique is added to the proposed ER method to improve the learning process in the current task with varying memory sizes. Experimental results on Pascal VOC show that the proposed ER method provides acceptable results for continual learning. Specifically, the proposed ER method has achieved mAP of 65.5% and 68.9% in 10 + 10 and 19 + 1 classes scenario, respectively, higher than the state-of-the-art method. Nevertheless, continual learning for object detection still desires more improvement. A further evaluation that integrates with thoroughly extensive experiments on improving the continual learning process is expected to be performed for future study. Furthermore, the proposed ER method has alleviated the problem of catastrophic forgetting. However, enough improvements are needed to reduce the use of memory size to the minimum level while maintaining classes accuracy. 

## Figures and Tables

**Table 1:** The task distribution between three different training scheme.

Task Distribution	Task $l^{\left[0\right]}$	Task $l^{\left[1\right]}$
10 + 10	2868 images (4852 objects)	3252 images (7756 objects)
19 + TV	4931 images (7918 objects)	256 images (324 objects)
19 + Person	4112 images (3552 objects)	2008 images (4690 objects)

**Table 2:** Parameters for training the model.

Parameters	Value
Momentum	0.9
Batch size	32
Epoch	100
Weight decay	0.0005
Initial learning rate	0.002
Final learning rate	0.0005
Image size	416 × 416

**Table 3:** Comparison of the proposed ER method with state-of-the-art methods for 19 + 1 classes scenario. Best results are written in bold number.

Methods	Aero	Bike	Bird	Boat	Bottle	Bus	Car	Cat	Chair	Cow	Din. Table	Dog	Horse	M Bike	Person	Plant	Sheep	Sofa	Train	TV	mAP at 0.5
All data	76.5	84.4	68.9	59.3	55.6	82.1	85.9	79.9	55.4	73.7	67.8	79.9	85.2	84.4	83.1	46.1	71	70.3	82.1	71.7	73.2
KD [1]	50.2	53.0	29.8	27.5	13.3	55.4	67.2	45.5	13.3	36.9	34.2	38.7	60.7	56.3	38.5	10.5	33.8	33.0	55.6	15.8	38.4
DMC [17]	**77.2**	67.8	**63.5**	46.5	50.2	70	79.3	80.3	43.5	65.5	0.2	71.1	74.6	71.5	78.2	39.6	66.6	34.8	79.2	42.4	60.1
GEM [15]	66.6	72.6	54.9	48.1	46.7	72.5	80.5	68.7	37.9	66.3	48.4	62.5	81	72.5	67.3	33.5	68	45.8	76.9	28.4	60
ER*	73.2	83	62.9	56.3	**53.7**	81.1	84.9	77.7	48.4	**69.1**	**63.6**	**78.5**	**85.7**	**82.6**	**80.9**	**43.3**	67.7	60.8	79.6	39.7	68.6
ER**	76.7	**83.1**	62.5	**56.8**	53.2	**83.8**	**85**	**78.1**	**50.1**	68.6	62.1	77.4	85.6	79.7	80.7	42.8	**70.6**	**66.2**	**81.1**	33.3	**68.9**
ER***	74.3	79.5	59.5	55.3	49.5	78.9	83.5	77.1	45.2	66	57.6	73.9	85.5	80.3	78.3	40.1	68	58.1	78.7	**44.7**	66.7

ER* method without augmentation before uploading to memory (2500), ER** method with augmentation after uploading to memory (2500), ER*** method with augmentation after uploading to memory (1000).

**Table 4:** Comparison of the proposed ER method with state-of-the-art methods for 10 + 10 classes scenario. Best results are written in bold number.

Methods	Aero	Bike	Bird	Boat	Bottle	Bus	Car	Cat	Chair	Cow	Din. Table	Dog	Horse	M Bike	Person	Plant	Sheep	Sofa	Train	TV	mAP at 0.5
All data	76.5	84.4	68.9	59.3	55.6	82.1	85.9	79.9	55.4	73.7	67.8	79.9	85.2	84.4	83.1	46.1	71	70.3	82.1	71.7	73.2
KD [1]	10.8	10.2	36.1	9.6	6.3	5.4	14.3	41.7	3.8	19.4	42.5	53.5	76.5	66.8	57.9	27.8	60.4	50.4	72.2	46	35.5
DMC [17]	56.6	46.5	48.7	25.7	45.5	62.9	71.5	65.7	36.8	52.4	0.6	60.1	71.2	58.2	73	40.7	58.9	12.6	64	66.6	50.9
GEM [15]	3.1	3.4	3.6	6.1	19.7	6.1	58.6	9.5	**55.7**	14.2	0.7	7.6	4.2	2.9	23.5	0.2	1.2	2.6	3.4	0.2	11.2
ER*	67.2	74.1	47.3	44.4	44.7	71	80.1	64.5	36.9	57.1	42.2	54.9	78.5	66.7	63.5	26.3	51.4	45	68.9	52.1	56.9
ER**	**75.5**	**82**	**58.9**	**51.5**	**49.5**	**78.7**	**84.8**	**74.1**	47.2	**65.1**	61	**65.4**	78.7	**76.6**	74.6	34.6	60.6	58.4	**77.8**	61.9	**65.5**
ER***	66.7	68.5	38	45.7	40.7	69.8	81.4	60.3	30.9	52.3	**67.4**	60.4	**81.1**	**76.6**	**79.6**	**43.9**	**61.1**	**61.3**	77.4	**64.7**	61.4

ER* method without augmentation before uploading to memory (2500), ER** method with augmentation after uploading to memory (2500), ER*** method with augmentation after uploading to memory (1000).

**Table 5:** Comparison of the proposed ER method with the state-of-the-art methods for 19 + 1 classes scenario when the incremental class person. Best results are written in bold number.

Methods	Aero	Bike	Bird	Boat	Bottle	Bus	Car	Cat	Chair	Cow	Din. table	Dog	Horse	M bike	TV	Plant	Sheep	Sofa	Train	Person	mAP at 0.5
Classes (1–19)	75.8	83.9	66.7	58.0	53.1	81.8	85.4	78.3	53.7	75	66.5	78.5	84.6	83	82.7	43.5	71.4	69.4	81.2	-	68.8
Class (20)	-	-	-	-	-	-	-	-	-	-	-	-	-	-	-	-	-	-	-	64.1	64.1
GEM [15]	61.7	65.9	42.6	37.2	34.7	65.2	68.4	59.6	30.9	50.3	45.1	54.9	65.6	65.2	52.7	23.8	52.5	50.1	62.1	50.3	51.9
ER	76.7	81	58	53.2	50.7	81	84.4	75	47	67.6	62.4	71.8	82.3	79.7	67.7	38.8	67.4	64.9	80.8	51.2	67.1

**Table 6:** Comparison of 10 + 10 classes with different memory sizes.

Methods	mAP(1–10)	mAP(10–20)	mAP
ER** (500)	42.5	64.4	53.5
ER** (1000)	55.4	67.4	61.4
ER** (2500)	66.3	65.2	65.7
ER** (5000)	66.7	65.0	65.8

**Table 7:** The number of images and objects for training and testing the proposed method on the ITRI-DriveNet-60 dataset.

Dataset	Four-Wheel Vehicle	Rider	Two-Wheel Vehicle	Person
Train	3099 images (17,879 objects)	652 images (1911 objects)	1146 images (5697 objects)	726 images (1053 objects).
Test	644 images (3116 objects)	117 images (311 objects)	195 images (881 objects)	128 images (202 objects).
Total	3643 images (20,995 objects)	769 images (2222 objects)	1341 images (6578 objects)	854 images (1255 objects).

**Table 8:** Test results of ER trained on ITRI DriveNet-60 dataset in 1 + 3 class continual scheme.

Methods	Four-wheel vehicle	Rider	Two-wheel vehicle	Person	mAP
Normal Training	89.7	72	80.2	66.9	77.2
$l^{\left[0\right]}$ Training	91.3	-	-	-	91.3
$l^{\left[1\right]}$ Training	85.2	77.3	80.2	65.6	77.1

**Table 9:** Training time for single iteration of the proposed ER method and prior works.

Methods	Proccess	Time (ms)
Normal Training	1× inference + 1× backpropagation	2380
KD [1]	2× inference + 1× backpropagation	2660
DMC [17]	3× inference + 1× backpropagation	2940
GEM [15]	2× (inference + backpropagation)	4760
ER	1× inference + 1× backpropagation + 1× augmentation	2580

Inference time = 280 ms, backpropagation time = 2100 ms, augmentation time = 200 ms.

**Table 10:** Advantages and disadvantages of the proposed ER method with state-of-the-art methods.

Methods	Advantages	Disadvantages
KD [1]	No memory required and faster training time	Auxiliary data required.
DMC [17]	No memory required	Three models required to train and auxiliary data required
GEM [15]	No memory and no auxiliary data required	Higher training time and lower performance
ER	No auxiliary data and faster training time	Memory required

## Abbreviations

The following abbreviations are used in this manuscript:

ADV	Autonomous Driving Vehicles
AP	Average Precision
BCE	Binary Cross Entropy
DMC	Deep Model Consolidation
ER	Experience Replay
Faster R-CNN	Faster Region Convolution Neural Networks
GEM	Gradient Episodic Memory
GIOU	Generalized Intersection Over Union
ITRI	Industrial Technology Research Institute
KD	Knowledge Distillation
YOLO	You Only Look Once
